# Concomitant presentation of thrombotic thrombocytopenic purpura, immune thrombocytopenia, and autoimmune hemolytic anemia in a patient with newly diagnosed systemic lupus erythematosus 

**DOI:** 10.5414/CNCS111193

**Published:** 2023-12-12

**Authors:** Lina Bruns, Linus Völker, Robert Klamroth, Martin K. Kuhlmann, Wolfram J. Jabs

**Affiliations:** 1 Department of Nephrology, Vivantes Clinic in Friedrichshain, Berlin,; 2 Department II of Internal Medicine and Center for Molecular Medicine Cologne (CMMC), University Hospital Cologne, Cologne, and; 3 Department of Angiology and Hemostaseology, Vivantes Clinic in Friedrichshain, Berlin, Germany

**Keywords:** acquired thrombotic thrombocytopenic purpura, autoimmune hemolytic anemia, immune thrombocytopenia, systemic lupus erythematosus, thrombocytopenia

## Abstract

Thrombocytopenia is always of concern when encountered in emergency settings. We report a case of a 29-year-old women in whom a unique constellation of hematological disorders occurred. The patient had been diagnosed with idiopathic immune thrombocytopenia (ITP) in 2007, with a history of several thrombocytopenic flares. She now presented with homonymous hemianopia accompanied by thrombocytopenia and microangiopathic hemolytic anemia (MAHA) and was soon after diagnosed with a posterior stroke. Symptoms were more reminiscent of acquired thrombotic thrombocytopenic purpura (aTTP) rather than ITP. Immediate treatment with plasma exchange and caplacizumab curtailed MAHA, and progressive ischemic disease was averted. ADAMTS-13 testing confirmed the diagnosis of immune-mediated aTTP. Repeated testing for ITP, however, also showed IgG-loaded thrombocytes with the former known anti-GPIIb/IIIa specificity. Furthermore, autoimmune hemolytic anemia (AIHA) could be detected by direct antiglobulin test showing IgG and complement loading of the patient’s erythrocytes. The autoimmune background of all three entities suggested an underlying systemic disease. Indeed, systemic lupus erythematosus (SLE) serology was strongly positive allowing for the diagnosis of SLE. ITP and AIHA as well as aTTP can be secondary to SLE, but emergence of all three disorders has not been reported at the same time.

## Introduction 

Acquired thrombotic thrombocytopenic purpura (aTTP) is a rare disease, which combines severe thrombocytopenia, microangiopathic hemolytic anemia (MAHA), and ischemic end-organ damage. A severe deficiency in A disintegrin and metalloprotease with thrombospondin-1-like domains (ADAMTS)-13, which cleaves ultra-large von Willebrand factor (ULvWF)-multimers thus preventing capillary microthrombosis has been acknowledged as the main pathogenic cause of this rare disease [1]. The discovery of the pathogenic mechanism behind aTTP made it possible to develop new therapies for this potentially fatal disease, namely caplacizumab, a nanobody that interrupts the interaction of ULvWF with platelets [2, 3]. In adults, the disease is mainly autoimmune acquired by idiopathic formation of autoantibodies against the ADAMTS-13 protease that inhibit spontaneous cleavage of ULvWF multimers. It can also be of secondary nature in the context of systemic diseases, like systemic lupus erythematosus (SLE), HIV infection, or malignancies [4]. On the contrary, immune thrombocytopenia (ITP) as well as autoimmune hemolytic anemia (AIHA) result from direct autoantibody formation against blood cell surface antigens [5, 6]. Both entities also can occur in the course of SLE, but the combination of all three entities (ITP, AIHA, aTTP) has not been reported yet. We herein report this unique setting of pathologies and describe our diagnostic approach and targeted therapies combining caplacizumab, rituximab, and belimumab. The patient gave written informed consent for publication of her case. 

## Case report 

The 29-year-old female patient was transferred to our hospital with a history of headache and a 4-day-old compensated left homonymous hemianopia. During the preceding weeks, she occasionally suffered from burning sensations in the left chest, which by the time of referral had stopped. The patient’s medical history was remarkable for ITP, firstly diagnosed in 2007 with anti-GPIIb/IIIa-antibodies (Ab) specificity. At last, the patient received romiplostim in December 2020 owing to refractory disease. Physical examination revealed petechiae on the lower abdomen. Initial laboratory results showed severe thrombocytopenia (12/nL), anemia (hemoglobin 10.4 g/dL), elevated creatinine (1.09 mg/dL), troponine T (492 ng/L; ref. < 14), and lactate dehydrogenase (LDH) (548 U/L). We strongly suspected aTTP rather than her known ITP as cause for the acute medical condition and therefore tested activity of ADAMTS-13 (activity ELISA, Technoclone, Vienna, Austria). As urgent treatment was warranted, but results of the ADAMTS-13 testing were delayed, we resorted to the PLASMIC score, which was 7/7, indicating a high probability for severe ADAMTS-13 deficiency [7], before initiating daily therapeutic plasma exchange (PEX) against fresh frozen plasma and caplacizumab therapy. Furthermore, immunosuppression with prednisolone (100 mg/day) was started [8, 9]. In the meantime, schistocytes were detected in peripheral blood smears (2.2 ‰) as a marker of microangiopathic hemolytic anemia (MAHA). Results of ADAMTS-13 testing showed a markedly reduced activity of the protease (1.7%; ref. 50 – 110%) concurrent with moderately increased concentrations of anti-ADAMTS-13 Ab (31.2 U/mL; ref. < 16) confirming the diagnosis of immune-mediated aTTP. 

**Figure 1.**
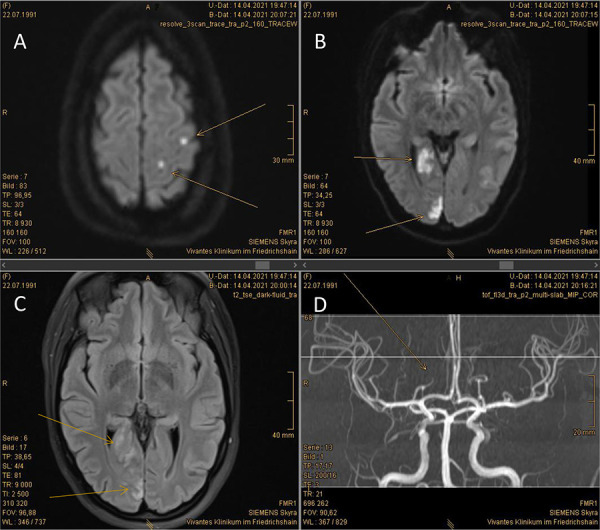
MRI scan of the brain owing to left homonymous hemianopia. A: Diffusion-weighted magnetic resonance imaging (DWI) showing at least two acute non-embolic microangiopathic infarctions of the left cerebral hemisphere (arrows). B: DWI sequence showing an acute territorial infarction of the right posterior cerebral artery (arrows). C: FLAIR sequence of the brain confirming right-sided posterior infarction (arrows). D: MR angiography of the intracranial arteries showing a peripheral thrombosis of the right posterior cerebral artery (arrow).

Because of the hemianopia, a cranial MRI scan was ordered, which showed an acute thrombotic infarction of the right posterior cerebral artery along with two minor infarctions of the left cerebral hemisphere (Figure 1). Troponin T levels rose to a maximum of 612 ng/L; concurrent ST changes and the aforementioned atypical angina pectoris symptoms led us to diagnosis of non-ST elevation myocardial infarction type II, most likely due to the ongoing thrombotic microangiopathy (TMA). Furthermore, the patient showed signs of acute kidney injury stage 1 (plasma creatinine 1.09 mg/dL), another manifestation of the presumed underlying TMA. 

Knowing the medical history of the patient, the diagnostic work-up was completed by another testing of antibodies against thrombocytes, confirming presence of thrombocyte-bound anti-GPIIb/IIIa Ab (direct monoclonal antibody immobilization of platelet antigens (MAIPA)). Similarly, Coombs testing was performed (direct antiglobulin test) (DAT)), which came back positive as well, showing erythrocyte loading with complement-binding autoantibodies of undeterminable specificity. 

After 4 days of treatment with PEX and caplacizumab, platelet counts rose to 184/nL. PEX was stopped, daily caplacizumab was continued, and immunosuppression with rituximab (1,000 mg) was added to prednisolone [8, 9]. The patient was continued on caplacizumab and prednisolone medication and was discharged on day 9. A second dose of rituximab was scheduled 14 days after the first dosage. Creatinine and troponin T levels had normalized in the meantime, but homonymous hemianopia remained. 

Six days after discharge, the patient was re-admitted with fever, non-productive cough, rising CRP (55.1 mg/L), and platelet counts of 24/nL. Despite ongoing treatment with caplacizumab and prednisolone, relapse of aTTP was considered due to increasing levels of LDH, but rapidly excluded because of the absence of MAHA (lack of schistocytes, increased haptoglobin level). Thus, we suspected another ITP flare triggered by the respiratory tract infection. Blood cultures were sampled, and the patient was started on a calculated antibiotic therapy with tazobactam/piperacillin and roxithromycin along with a pulse therapy of prednisolone (250 mg/day). After 3 days of treatment, the patient’s general condition improved, and platelet counts recovered to 165/nL. Prednisolone (50 mg/day) and caplacizumab (10 mg/day) were continued upon discharge. 

Upon follow-up, serology for SLE was performed, demonstrating highly positive titers of anti-nuclear Ab (ANA, 1 : 5,120). In addition, ELISA testing showed markedly increased levels of anti-dsDNA Ab (189.8 U/mL). No evidence of anti-phospholipid syndrome (APS) was obtained, as tests for lupus anticoagulant, anti-cardiolipin, and anti-β_2_-glycoprotein Ab were negative. Urine analysis showed evidence of lupus nephritis with a glomerular proteinuria of 4.8 g protein/g creatinine and mild hematuria. Kidney biopsy was performed demonstrating membranous lupus nephritis (class V) thus confirming the diagnosis of SLE with an ACR/EULAR score of 18 [10]. 

**Figure 2.**
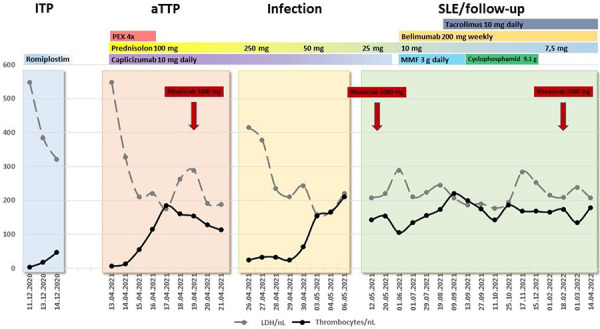
Clinical course demonstrating four different phases of thrombocytopenia, underlying etiologies and chosen therapies. The clinical course of the patient could be grouped into four different phases dominated by thrombocytopenia: The first phase (light blue) represented her last idiopathic immune thrombocytopenia (ITP) flare in December 2020, resulting in romiplostim therapy due to refractory disease. At that timepoint, autoimmune hemolytic anemia (AIHA) as well as microangiopathic hemolytic anemia (MAHA) were not known to exist, but increased lactate dehydrogenase levels may point towards a missed hemolysis. The second phase started in April 2021 (orange) and was dominated by the newly diagnosed acquired thrombotic thrombocytopenic purpura (aTTP), introducing plasma exchange and caplacizumab as well as rituximab in the patients’ therapeutic regimen. The third thrombocytopenic phase (yellow) superimposed aTTP and was due to overt upper respiratory tract infection with blastic cells in peripheral blood smears. It was mainly resolved by antibiotic treatment and increased doses of prednisolone. The fourth phase (light green) reflected the phase of SLE diagnosis and treatment as well as aTTP follow-up with stable thrombocyte counts and resolved ITP, AIHA, and MAHA.

After administration of a second dose of rituximab on day 38, ADAMTS-13 activity slowly returned to normal with undetectable anti-ADAMTS-13 Ab levels (Table 1). Caplacizumab was stopped on day 45, and the immunosuppressive regimen was adapted to the diagnosis of SLE (Figure 2). Treatment of SLE became challenging over the next months, as lupus nephritis class V developed into a life-threatening nephrotic syndrome not responding to standard therapy consisting of mycophenolate mofetil (MMF), belimumab, and prednisolone. Therefore, tacrolimus was added to the therapeutic regimen, and later on MMF was substituted by IV boli of cyclophosphamide. A third dose of rituximab was administered due to toxic side effects of cyclophosphamide (severe alopecia), finally resulting in partial remission of nephrotic syndrome with preserved estimated glomerular filtration rate of 122 mL/min. 

There was no recurrence of detectable hemolysis, and thrombocyte counts as well as ADAMTS-13 activity remained stable throughout a 1-year follow-up. Upon intensified immunosuppression, DAT became negative, and thrombocytes no longer displayed loading with thrombocyte-specific Ab (Table 1). 

## Discussion 

This case report describes the concomitant presentation of three hematological disorders of a young SLE patient, namely aTTP, ITP, and AIHA, that have never been described in one patient at the same time. A recent review summarized 28 patients with immune-mediated aTTP in patients with and without SLE at a single center. TTP was regarded SLE-related in 10 patients and primary in 18 patients. SLE-related aTTP was considered more favorable regarding clinical remission, acute kidney injury, and mortality [11]. 

The sequential occurrence of aTTP after ITP or vice versa is well recognized in the literature [12, 13, 14]. It has been described in HIV infected patients [15, 16, 17] and in patients with primary Sjögren’s syndrome or post-partum [18, 19]. None of these cases, however, proved the autoimmune background of the ITP case, as IgG loading of thrombocytes was never investigated. In our patient, ITP was diagnosed early in 2007, demonstrating thrombocyte-specific IgG with anti-GPIIb/IIIa specificity first in 2011. The acute presentation of a posterior stroke in the context of MAHA now lead to the additional diagnosis of immune-mediated aTTP with severe ADAMTS-13 deficiency due to inhibitory ADAMTS-13 Ab. Nonetheless, thrombocytes of the patient again displayed positive thrombocyte-specific Ab at the same time. Moreover, erythrocytes of the patient also showed an intense loading of complement-binding Ab of undeterminable specificity by DAT in the context of proven MAHA with schistocytes of 2.2 ‰ in peripheral blood smear. This presentation seems to be unique. It is likely that the hemolysis seen in our patient was mainly due to the newly diagnosed MAHA; however, LDH levels already had been elevated in 2020, opening the possibility of misdiagnosed AIHA at an earlier timepoint. 

Figure 2 outlines the patient’s clinical course during the last thrombocytopenic episodes and highlights the presumed etiologies and the chosen therapies. As mentioned before, reasons for elevated LDH levels seen in 2020 had not been investigated. They might be attributable to undetected AIHA or missed MAHA as well. In 2020, the patient was started on romiplostim due to increasing episodes of thrombocytopenic flares stabilizing thrombocyte counts between 70 and 119/nL. The clinical course of the patient with a persistent thrombocytopenia despite treatment with romiplostim and an elevated LDH raises the question whether aTTP was already present in the last months before aTTP diagnosis in April 2021. However, no further diagnostic tests had been performed at that timepoint to exclude this possibility, and the patient remained stable with no clinical signs of TMA. 

In April 2021, diagnosis of immune-mediated aTTP was unambiguous due to undetectable ADAMTS-13 activity. Nevertheless, the patient displayed another thrombocytopenic flare 12 days after starting caplacizumab during an episode of upper respiratory tract infection and fever. Thrombocytopenia was accompanied by increased levels of LDH, but also increased haptoglobin and with no evidence of ongoing MAHA and thrombocytes proved to be loaded with anti-GPIIb/IIIa Ab. However, ADAMTS-13 activity was still undetectable at that timepoint with increasing levels of anti-ADAMTS-13 Ab. Therefore, thrombocytopenia during this fever episode could be explained by ITP responding promptly to glucocorticoid treatment as well as by aTTP despite sufficient therapy with caplacizumab. 

The presentation of a thrombotic event, namely posterior stroke, in the presence of ITP, which is characterized predominately by bleeding episodes, called for another, more prothrombotic disease. Besides aTTP, APS as well as heparin-induced thrombocytopenia (HIT) share the hallmark of thrombocytopenia and the immune mediated pathogenesis. In all three entities, platelet and endothelial cell activation occurs, resulting in a prothrombotic state and an increased risk of thrombosis [20]. As the patients’ medical history easily ruled out HIT, APS could also be excluded. Besides our patient, several other case presentations documented the combination of at least two of the four aforementioned diseases in the same patient, illustrating the concept of a common autoimmunity background [20]. This concept appears more than true in our patient with SLE as the underlying prototypic autoimmune disease. 

Diagnosis of SLE was delayed in our patient due to the lack of other clinical signs like cutaneous lupus, arthritis, or serositis [10] and lack of specific autoantibody testing. It is conceivable that SLE was already present years before, and that continued immunosuppression might have averted the development of progressive SLE with aTTP and lupus nephritis. In fact, current treatment of SLE lead to negative ADAMTS-13 Ab, negative DAT and MAIPA for thrombocyte-specific Ab; ANA titers dropped to 1 : 640 with decreasing anti-dsDNA Ab and preserved kidney function. 

In summary, concomitant presentation of ITP, AIHA, and MAHA due to immune-mediated aTTP might occur in SLE patients. A positive DAT is not sufficient to rule out episodes of aTTP in selected SLE or ITP patients in whom medical history points towards thrombotic rather than bleeding events. 

## Acknowledgment 

We are grateful to Prof. H.-J. Wagner, head of department of radiology, Vivantes Clinic Friedrichshain, Berlin, Germany, for providing and interpreting MR imaging of the cerebrum of the presented case (Figure 1). 

## Funding 

The authors declare that no funding applies to this study. 

## Conflict of interest 

The authors declare that there is no conflict of interest. 

**Table 1.** Course of ADAMTS-13 activity, systemic lupus erythematosus autoantibodies, direct antiglobulin test, and MAIPA.

	11.05.2011	13.04.2021	22.04.2021	28.04.2022	05.05.2021	12.05.2021	01.06.2021	01.07.2021	19.08.2021	09.09.2021	01.03.2022
ADAMTS-13 activity (%; 50 – 110)		2	< 1	< 1	4	28	32	39	62	82	99
ADAMTS-13 Ab (U/mL; < 16)		31	40	56	16	8	15	8	7	5	5
ANA (titer)					1 : 5,120	1 : 5,120	1 : 10,240	1 : 2,560	1 : 2,560	1 : 2,560	1 : 640
dsDNA Ab (ELISA; U/mL; < 20.0)					189.8	189.3	> 200	137.3	96.3	98.4	74.9
Coombs test (DAT)											
Anti-IgG		+	+++								–
Anti-C3d		+	++								–
Thrombocyte-specific Ab (MAIPA)											
GP IIb/IIIa	+		+								–
GP Ib/IX	–		+								–
GP Ia/IIa	–		–								–

ADAMTS = A disintegrin and metalloprotease with thrombospondin-1-like domains; Ab = antibody; ANA = anti-nuclear antibody; dsDNA = double-stranded DNA; DAT = direct antiglobulin test; IgG = immunoglobulin G; C3d = complement 3d; MAIPA = monoclonal antibody immobilization of platelet antigens; GP = glycoprotein.
